# AP-3 and Rabip4’ Coordinately Regulate Spatial Distribution of Lysosomes

**DOI:** 10.1371/journal.pone.0048142

**Published:** 2012-10-29

**Authors:** Viorica Ivan, Emma Martinez-Sanchez, Livia E. Sima, Viola Oorschot, Judith Klumperman, Stefana M. Petrescu, Peter van der Sluijs

**Affiliations:** 1 Department of Cell Biology, University Medical Center Utrecht, Utrecht, The Netherlands; 2 Department of Molecular Cell Biology, Institute of Biochemistry of the Romanian Academy, Bucharest, Romania; Purdue University, United States of America

## Abstract

The RUN and FYVE domain proteins rabip4 and rabip4’ are encoded by *RUFY1* and differ in a 108 amino acid N-terminal extension in rabip4’. Their identical C terminus binds rab5 and rab4, but the function of rabip4s is incompletely understood. We here found that silencing *RUFY1* gene products promoted outgrowth of plasma membrane protrusions, and polarized distribution and clustering of lysosomes at their tips. An interactor screen for proteins that function together with rabip4’ yielded the adaptor protein complex AP-3, of which the hinge region in the β3 subunit bound directly to the FYVE domain of rabip4’. Rabip4’ colocalized with AP-3 on a tubular subdomain of early endosomes and the extent of colocalization was increased by a dominant negative rab4 mutant. Knock-down of AP-3 had an ever more dramatic effect and caused accumulation of lysosomes in protrusions at the plasma membrane. The most peripheral lysosomes were localized beyond microtubules, within the cortical actin network. Our results uncover a novel function for AP-3 and rabip4’ in regulating lysosome positioning through an interorganellar pathway.

## Introduction

Lysosomes are dynamic membrane-bound organelles that degrade macromolecules from the endocytic, secretory, and autophagic pathways [1], [2]. Lysosomes were traditionally appreciated for their degradative function, but it is now clear that they serve more complex roles like plasma membrane repair, and as intracellular signaling platforms [1], [2], [3]. Melanocytes and hemopoietic cells contain lysosome-related organelles (LRO), also known as secretory lysosomes. In addition to housekeeping content, LRO contain a complement of distinct molecules for cell type specific functions. The significance of LRO is dramatically highlighted by the severe human diseases caused by mutations in genes regulating their functions [4], [5].

Lysosomes and LRO undergo motor-directed bidirectional transport along microtubules. Kinesin-1 and kinesin-2 transport lysosomes towards microtubule plus-ends [6], [7], [8], whereas retrograde movement requires the dynein-dynactin motor complex [9], [10], [11]. The relative density of opposing motor proteins is thought to determine the net direction of motility and position of lysosomes in the cytoplasm. The mechanisms for the recruitment of motor proteins to membranes are incompletely understood. Several proteins have been implicated in regulating lysosome positioning. Amongst them is the rab7-RILP-ORP1L complex that together with betaIII spectrin recruits dynein to late endosomes [9], [11], [12], [13]. The small GTPse Arl8 and PLEKHM2 are needed for kinesin-1 accumulation on lysosomes and distribution of lysosomes in the cell periphery [14].

Membrane proteins reach their steady state distribution via transport carriers that shuttle cargo between organelles. The requisite sorting processes in post Golgi compartments are executed by cytoplasmic cargo selectors, such as adaptor protein complexes (AP), in conjunction with accessory proteins and phosphatidylinositols. Five heterotetrameric adaptor complexes, AP-1 to AP-5, are presently known [15]. Their localization to distinct intracellular membrane domains is an important factor in establishing specificity in the formation of transport carriers. AP-1 and AP-2 are necessary for normal embryonic development, but the requirement for other AP complexes is less stringent. AP-3 occurs in two forms that share the common δ and σ3 subunits and diverge with respect to β3 and μ3 subunits. Ubiquitously expressed AP-3A contains μ3A and β3A, while brain specific AP-3B has the corresponding β3B and μ3B subunits [16]. Hermansky-Pudlak syndrome type 2 (HPS2) patients lack functional AP-3A and suffer from pigmentation defects, bleeding disorders, and immune deficiency [17], [18], traits that are phenocopied in the pearl mouse with a mutation in β3A [19]. AP-3 localizes to budding profiles evolving from early endosome-associated tubules where it defines an exit pathway for tyrosinase to melanosomes and for lysosome-associated membrane proteins (LAMPs) to lysosomes [20], [21]. Loss of AP-3A redirects LAMPs to the cell surface [17], mislocalizes the late endosomal/lysosomal v-SNARE Ti-VAMP to recycling endosomes [22], and causes accumulation of tyrosinase in early endosomes and in intralumenal vesicles of multivesicular bodies [20].

Rab GTPases are important regulators of endo-lysosomal transport [23]. They recruit effectors to relay the GTPase switch to downstream biological processes and in doing so create membrane heterogeneity in the endosomal network. Such microdomains serve as platforms for the different transport and signaling pathways [24]. We previously identified rabip4’, a long isoform of the RUN and FYVE domain-containing protein RUFY1, on endosomes where it interacts with rab5 and rab4 [25]. To understand rabip4’ function, we analyzed the intracellular pathways of several cargo proteins after rabip4s silencing and discovered that lysosomal proteins become specifically localized to peripheral protrusions. A search for rabip4’-interacting proteins yielded AP-3. We here also characterize the interaction between rabip4’ and AP-3 and document for the first time a role for both AP-3 and rabip4’ in the intracellular distribution of lysosomes via a pathway that is downstream of rab4.

## Results

### Rabip4’, Hrs, and EEA1 Define Partially Overlapping Endosomal Microdomains

**Figure 1:**
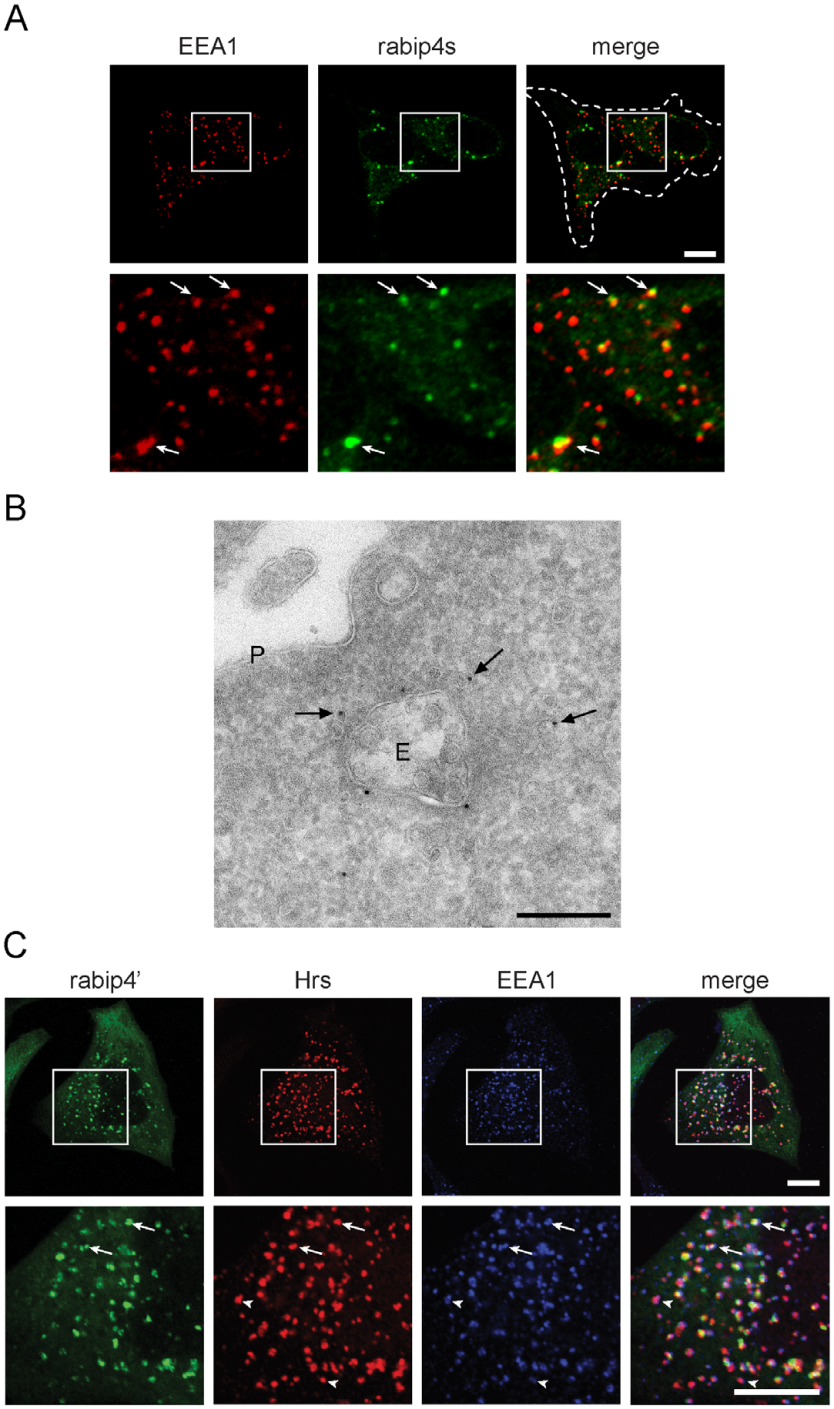
Rabip4’, EEA1, and Hrs partially colocalize on endosomes. HeLa cells were labeled for endogenous rabip4s and EEA1. Arrows denote co-distribution of the two FYVE domain-proteins. Bottom row represents a blow up of indicated areas. The dashed line marks the contour of cells. Scale bar is 10 µm (**A**). Ultrathin cryosections of HeLa cells expressing VSVG-rabip4’ were immunogold labeled with polyclonal anti-VSVG (10 nm gold particles). VSVG-rabip4’ localized to the endosomal vacuole (E), as well as to surrounding tubular-vesicular membrane profiles characteristic for recycling tubules (arrows). P is plasma membrane. Bar, 200 nm (**B**). HeLa cells transfected with GFP-rabip4’ and HA-Hrs were labeled for EEA1 (blue) and HA-Hrs (red), while rabip4’ was visualized by EGFP fluorescence. Bottom row represents an enlargement of the indicated areas. Arrows point to endosomes that contain rabip4’, EEA1, and Hrs, and arrowheads indicate endosomes devoid of rabip4’. Scale bar is 10 µm (**C**).

To better define the distribution of rabip4’, we raised an antibody against a common epitope of rabip4’ and rabip4/RUFY1, collectively called rabip4s. We found that endogenous rabip4’ had a similar distribution as the epitope-tagged protein [25], partially colocalizing with EEA1 (Figure 1A, inset). At the ultrastructural level, VSVG-rabip4’ was associated with the tubular and vacuolar portion of the early endosomal system (Figure 1B), in accord with its localization on the transferrin receptor (TfR) pathway [25]. We next compared the localization of GFP-rabip4’ relative to EEA1 and HA-Hrs by immunofluorescence microscopy. Triple labeling experiments showed partial colocalization between the three FYVE domain proteins (Figure 1C), suggesting that they are associated with overlapping, yet distinct endosomal domains. Approximately 90% of rabip4’-containing endosomes labeled for both EEA1 and Hrs. An endosomal population that contained EEA1 and Hrs but not rabip4’ could also be observed (Figure 1C, arrowheads). The partial segregation of rabip4’ with respect to the EEA1 and Hrs- populated domain correlates with different functions of the latter two in degradative pathways of the endo-lysosomal system [26], [27].

### Rabip4’ Localizes to Early Endosomes in a Rab5 and PI(3)P-dependent Manner

**Figure 2:**
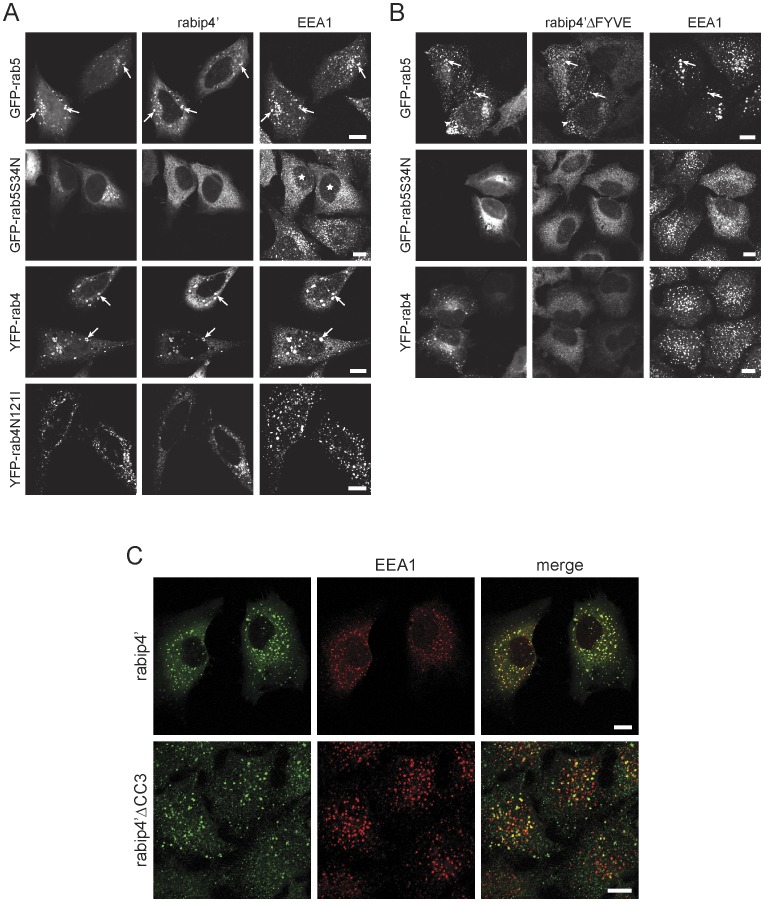
Rab5 is essential for endosome localization of rabip4’. HeLa cells expressing VSVG-rabip4’ and the indicated GFP/YFP-tagged rab5 and rab4 constructs were treated with wortmannin, and labeled for VSVG-rabip4’ and EEA1. Rabip4’ relocalized into the cytoplasm in cells expressing the GFP-rab5S34N mutant (asterisks). Arrows indicate colocalization between rabip4’, EEA1, and either of the GTPases (**A**). HeLa cells stably expressing VSVG-rabip4’ΔFYVE (aa 1–636) were transfected with the indicated GFP-rab5 and YFP-rab4 constructs, and labeled as above. Rab5 rescued the ΔFYVE mutant from the cytoplasm and relocalized it to endosomes that contained (arrows) or were devoid (arrowheads) of EEA1 (**B**). HeLa cells expressing VSVG-rabip4’ or VSVG-rabip4’ΔCC3 were labeled with antibodies against rabip4’ (rabbit) and EEA1 (mouse). The secondary antibodies were Alexa488-goat anti-rabbit and Cy3-goat anti-mouse. Scale bar is 10 µm (**C**).

Rabip4 was originally identified as a rab4 effector [28] and subsequent work showed that rabip4s also interact with rab5 [25] and rab14 [29]. A contribution of rab4 or rab5 to the recruitment of rabip4s, however, has not been defined. In the presence of wortmannin, VSVG-rabip4’ remained associated with enlarged endosomes that contained GFP-rab5 or YFP-rab4 and EEA1 (Figure 2A). Rabip4’ was, however, localized in the cytoplasm of cells expressing dominant negative rab5S34N that were treated with wortmannin. In contrast to the inactive rab5 mutant, co-expression of dominant negative YFP-rab4N121I did not affect rabip4’ or EEA1 (Figure 2A), showing that the rab5 interaction is required for endosomal localization of rabip4’. In accord, rab5 but not rab4 or rab5S34N relocated a cytoplasmic rabip4’ variant lacking the FYVE domain [25] to endosomes (Figure 2B). Therefore, the FYVE domain and the rab5 binding site are important and independent determinants for rabip4’ localization to EEA1-containing endosomal domains. In agreement with this, rabip4’ΔCC3 lacking the 3^rd^ coiled coil domain with the rab5 binding region [25] retained a punctate cytoplasmic distribution and colocalized with EEA1, but to a lesser extent than wild-type rabip4’ (Figure 2C), although it is also possible that overexpression of rabip4’ΔCC3 overcomes the need for rab5 binding.

### Rabip4’ Depletion Relocates CD63 and LAMP-1 to Plasma Membrane Protrusions

**Figure 3:**
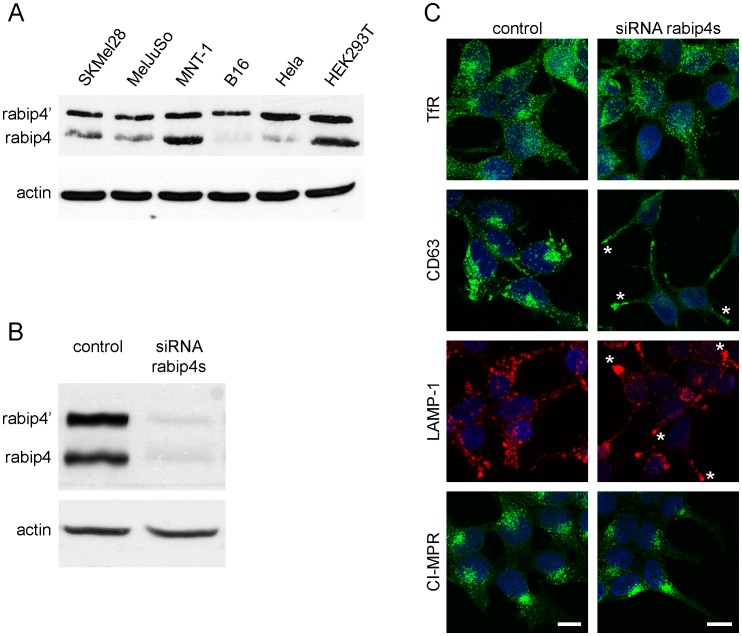
Rabip4’ functions in distribution of lysosomes. The indicated cell lines were screened for expression of rabip4s by Western blot (**A**). HEK293T cells were transfected with siRNA against rabip4s and three days later processed for Western blot with antibodies against rabip4s and actin as a loading control, followed by Alexa680-conjugated secondary antibody. siRNA induced an >90% reduction of both rabip4’ and rabip4 isoforms (**B**). In parallel, cells were labeled for immunoflourescence with monoclonal antibodies against CD63, LAMP-1, CI-MPR, and TfR. LAMP-1 was counterstained with Alexa 594-, while CD63, CI-MPR, and TfR with Alexa 488-labeled secondary antibodies. Nuclei were stained with DAPI. Images represent projections of confocal Z-stacks. Asterisks denote plasma membrane protrusions, enriched in CD63 and LAMP-1, induced by depletion of rabip4s. Scale bar, 10 µm (**C**).

We next explored the function of rabip4s through a combined knock-down in HEK293T cells that express high levels of endogenous rabip4s (Figure 3A). Western blot showed that siRNA treatment reduced rabip4s levels to <7% (Figure 3B). Depletion of rabip4s induced a very characteristic phenotype where CD63 and LAMP-1 redistributed from their central localization as seen in control cells into cellular projections where they frequently clustered at the tips of the protrusions (Figure 3C). Fifty-five percent of cells depleted for rabip4s showed this phenotype, as opposed to 15% of control cells. In addition, both CD63 and LAMP-1 containing organelles were slightly bigger in siRNA-treated cells. A similar observation was made for CD63 after rabip4s knock-down in the SKMel28 melanoma cell line (not shown). The phenotype observed in HEK293T cells was specific for CD63 and LAMP-1, because the distribution of CI-MPR was not grossly affected. We also analyzed the distribution of TfR in rabip4s-depleted cells. Although TfR-positive endosomes appeared somewhat enlarged and more scattered than in control cells, they were conspicuously absent from the protrusions containing CD63 and LAMP-1 (Figure 3C).

### Identification of a Rabip4’-interacting Protein Complex

**Figure 4:**
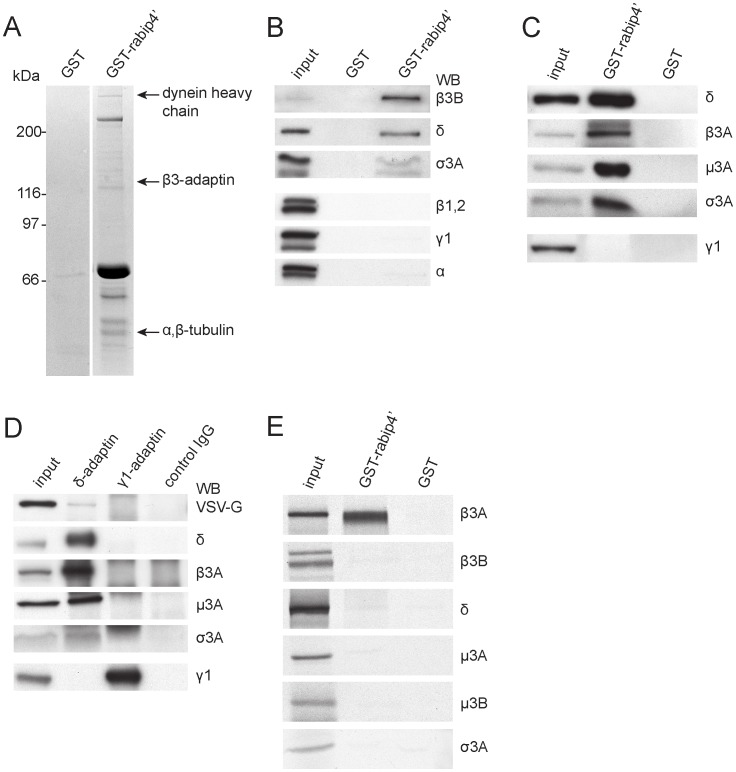
Rabip4’ interacts specifically and directly with AP-3. Immobilized GST-rabip4’ (aa 299–708) was incubated with brain cytosol. Bound proteins were resolved by SDS-PAGE and analyzed by tandem mass spectrometry, which yielded β3-adaptin as binding partner (**A**). Eluates were probed with antibodies against subunits of adaptor complexes showing specificity for AP-3 (**B**). GST-rabip4’ beads were incubated with detergent extracts from rescued *mocha* cells and bound proteins were analyzed by Western blot for the indicated AP-3 and AP-1 subunits. The ubiquitous AP-3 specifically interacted with rabip4’ (**C**). AP-3 was immunoprecipitated from lysates of HeLa cells expressing VSVG-rabip4’ and analyzed by Western blot with antibodies against VSVG and AP-3 subunits. Immunoprecipitation of AP-1 or a monoclonal antibody against HA (control IgG) did not co-immunoprecipitate VSVG-rabip4’ (**D**). GST-rabip4’ was immobilized on GSH beads and incubated with ^35^S-labeled AP-3 subunits. Rabip4’ interacted directly with AP-3 through the β3 subunit (**E**).

To understand how rabip4s control the distribution of CD63 and LAMP-1, we searched for interactors in a pull-down assay with GST-rabip4’(aa 299–708) and brain cytosol. We identified four peptides (LQVINLAAK, NVEVPEWTK, NASDLFPAVVK, and QLIVPSEQGGALSR) in the indicated ∼120 kDa band (Figure 4A), which led to its identification as β3B-adaptin and candidate partner of rabip4’. Other proteins that were identified included the heavy chain of cytoplasmic dynein and α, β-tubulin (Figure 4A). A Western blot with an antibody specific to β3B confirmed the mass spectrometry data (Figure 4B). β3B-adaptin is a component of the neuronal form of AP-3 that can be considered as a dimer consisting of β3-μ3 and δ-σ3 hemicomplexes. We next determined whether other AP-3 subunits also bound to GST-rabip4’(aa 299–708). Both δ- and σ3-adaptin were isolated on GST-rabip4’(aa 299–708), showing that rabip4’ bound to β3 in the context of the AP-3 complex (Figure 4B). The interaction was specific for AP-3 since neither the AP-1 subunits γ1 and β1 nor the large AP-2 α and β2 subunits bound to rabip4’. Brain also contains ubiquitously expressed AP-3A [30] that bound to rabip4’ as well in a pull-down with GST-rabip4’(aa 299–708) and detergent extracts of rescued *mocha* fibroblasts in which the δ subunit was re-introduced [31]. As shown in Figure 4C, we detected all the subunits of the ubiquitous AP-3 complex on rabip4’ beads. As expected from the binding data obtained with brain, we also found that AP-1 from rescued *mocha* cells did not interact with GST-rabip4’(aa 299–708). Thus, rabip4’ bound specifically to the generic and brain-specific forms of AP-3.

To extend this notion, we tested whether rabip4’ is present in a complex with AP-3 *in vivo* using a co-immunoprecipiation assay in HeLa cells expressing VSVG-rabip4’. An antibody against the δ subunit immunoprecipitated the other AP-3 subunits, as well as VSVG-rabip4’ (Figure 4D). The rabip4’*AP-3 interaction *in vivo* was specific since rabip4’ was not co-immunoprecipitated with AP-1 (γ1-adaptin) or with a control, non-relevant monoclonal antibody. To establish which of the four AP-3 subunits formed the link with rabip4’, we produced individual ^35^S-labeled AP-3 subunits and used them in a pull-down assay with GST-rabip4’. With the exception of β3A, none of the other subunits came down with GST-rabip4’ (Figure 4E). Even though we first found β3B in the mass spec of GST-rabip4’-bound proteins, it needs the context of the entire AP3 complex, perhaps because this large neuron-specific isoform is less stable than the other subunits. Since none of the other AP-3 subunits was enriched on the GST-rabip4’ column (Figure 4E), we concluded that AP-3 interacts through the β3 subunit with rabip4’.

### The Interaction of Rabip4’ with AP-3 is Mediated by the FYVE Domain

**Figure 5:**
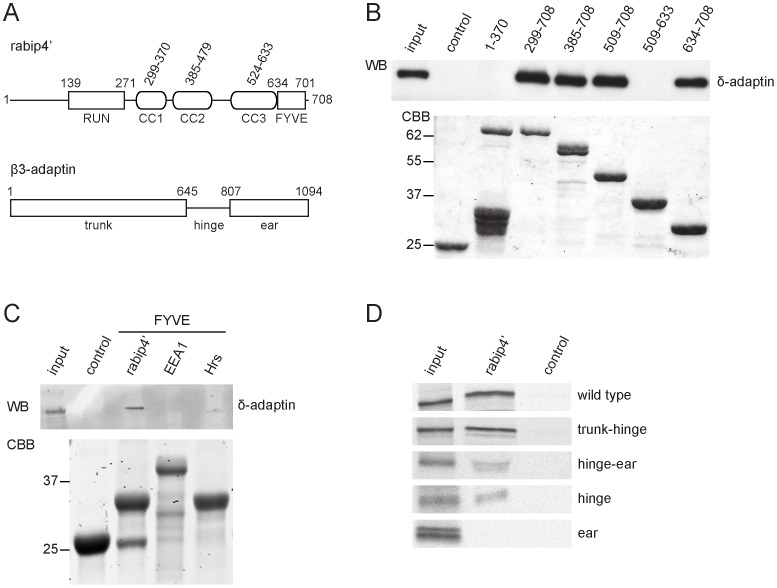
Interacting domains. Domain organization of rabip4’ and β3-adaptin (**A**). The binding domain of AP-3 on rabip4’ was determined with overlapping truncations of rabip4’ in a GST pull-down assay using detergent extracts from rescued *mocha* fibroblasts. Bound AP-3 was analyzed by Western blotting with antibodies against δ-adaptin. The AP-3 binding site was contained within the FYVE domain of rabip4’ (**B**) and is specific for rabip4’ since the FYVE domains of Hrs and EEA1 did not bind AP-3 (**C**). A pull-down assay with GST-rabip4’ and ^35^S-labeled Myc-tagged β3A truncations showed that the rabip4’ binding site is in the hinge region of β3A (**D**).

To gain insight into how rabip4’ and AP-3 interact, we next determined the binding requirements. Six overlapping rabip4’ truncation mutants (Figure 5A, B) were generated and used in a GST pull-down binding assay with a detergent extract prepared from rescued *mocha* cells. The shortest fragment that retained the ability to interact with AP-3 was the FYVE domain (Figure 5B), indicating that the binding site is located in the C terminus of rabip4’. Since the essential residues for PI(3)P binding are conserved between FYVE fingers, it became necessary to establish whether binding of AP-3 is a property of rabip4’ or FYVE proteins in general. GST pull-down assays with the FYVE domains of Hrs, EEA1, and rabip4’ showed that only the latter bound AP-3 (Figure 5C) and that non-conserved residues of the rabip4’ FYVE domain are essential for the interaction with AP-3. To map the region of β3A responsible for the interaction with rabip4’, we used ^35^S-labeled Myc-tagged versions of β3A deletion mutants (Figure 5A) in binding assays with GST-rabip4’(aa 299–708). Experiments with a series of truncations revealed that the hinge region is required and sufficient for the interaction (Figure 5D). Thus, the interaction between the two proteins required the FYVE domain of rabip4’ and the hinge interspaced between the trunk and the ear domains of β3A.

### AP-3 and Rabip4’ Colocalize on Endosomes

**Figure 6:**
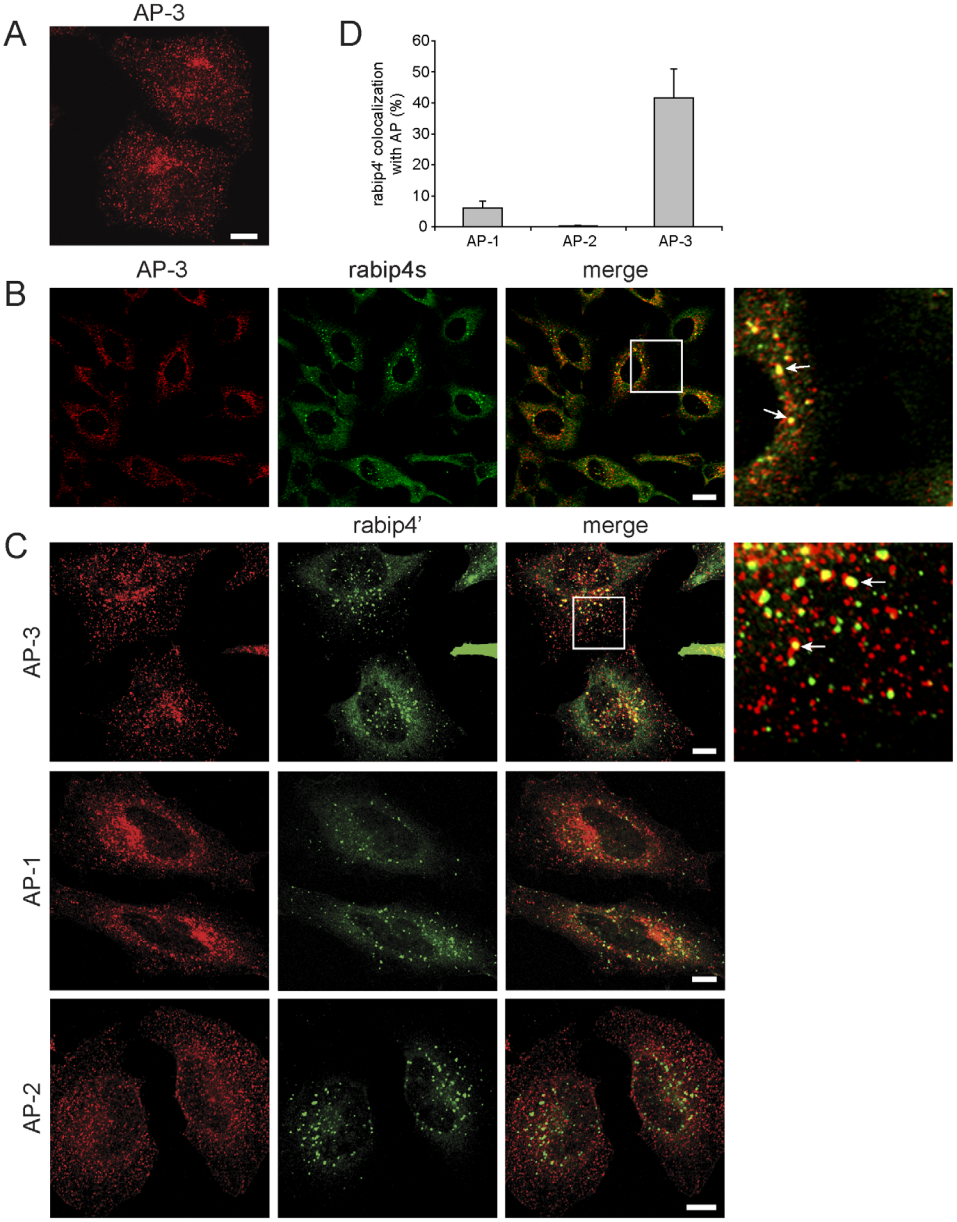
Rabip4’ and AP-3 colocalize on endosomes. HeLa cells were labeled with antibodies against δ-adaptin (**A**) or δ-adaptin (red) and endogenous rabip4s (green) (**B**). Scale bar is 10 µm (**A**) and 2.5 µm (**B**). HeLa cells expressing VSVG-rabip4’ were labeled with a rabbit antibody against rabip4’ (green) and monoclonal antibodies against the δ subunit of AP-3, the γ1 subunit of AP-1, and the α subunit of AP-2 (all in red). Scale bar is 10 µm. (**C**). Arrows in insets indicate structures on which AP-3 and rabip4s or VSVG-rabip4’ colocalize (**B and C**). Extent of colocalization between rabip4’ and the adaptor complexes (**D**).

We next examined the intracellular distribution of rabip4’ and AP-3 by confocal microscopy. In HeLa cells, we found AP-3 labeling on numerous small cytoplasmic structures scattered throughout the entire cell, with increased perinuclear density (Figure 6A). Double labeling of endogenous rabip4s and AP-3 revealed that a population of the AP-3 structures also contained rabip4s (Figure 6B, arrows in inset). To further characterize these, we expressed VSVG-rabip4’ and found that rabip4’ and AP-3 colocalized predominantly on endosomes located in the juxtanuclear area (Figure 6C, arrows in inset). Approximately 42% of membrane-bound rabip4’ colocalized with AP-3 (Figure 6C, D), while the distribution of AP-3 was similar in control (Figure 6A, B) and rabip4’-transfected cells (Figure 6C), showing that rabip4’ is not involved in the direct recruitment of AP-3 to endosomal membrane. The rabip4’*AP-3 structures are distinct from endosomes or endosomal domains to which AP-1 is localized and we found 10 times less colocalization of VSVG-rabip4’ with AP-1 (Figure 6C, D), while AP-2 did not co-distribute (Figure 6C, D). Thus, the rabip4’*AP-3 complex defines a specific domain of the endosomal network.

**Figure 7:**
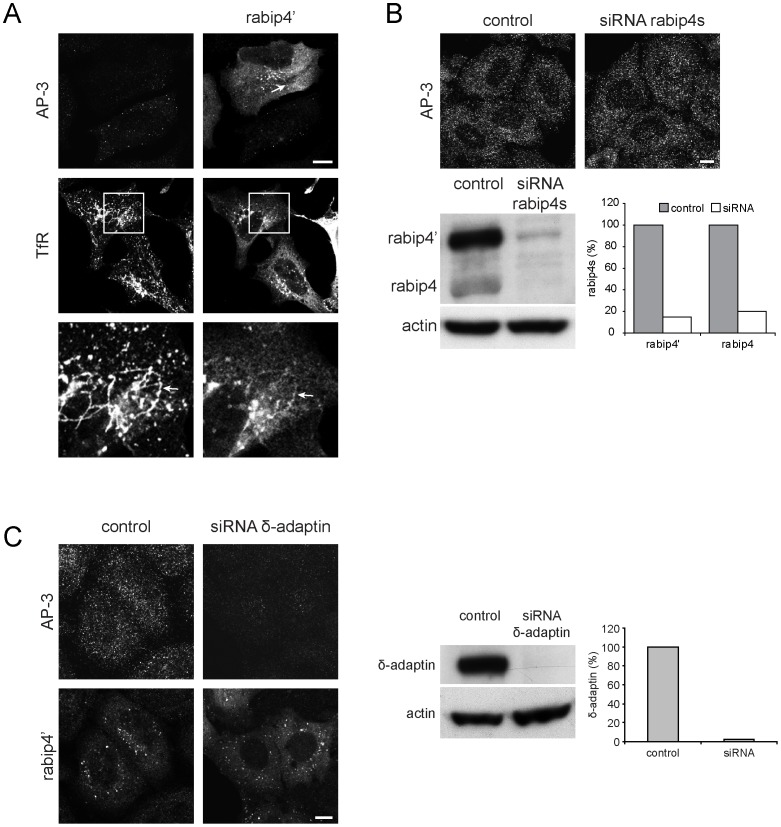
Rabip4’ localization does not require AP-3. VSVG-rabip4’-expressing HeLa cells were treated with 5 µg/ml BFA for 15 min at 37°C and stained with a rabbit antibody against rabip4’ and mouse anti-δ-adaptin or mouse anti-TfR, followed by Alexa568-anti-rabbit and Alexa488-anti-mouse IgG. Rabip4’ overexpression did not affect AP-3 sensitivity to BFA. Lower row represents insets of boxed areas. Arrows point to BFA-induced tubulation of rabip4’ and TfR (**A**). Rabip4s-directed siRNA oligos were transfected in HeLa cells for 3 days. AP-3 distribution was similar in both siRNA-transfected and control cells. Scale bar is 10 µm. Silencing was monitored by Western blotting and gave routinely 80–85% reduction of both rabip4’ and rabip4 isoforms (**B**). AP-3 siRNA oligos were transfected in HeLa cells for 3 days. Two days after siRNA treatment, cells were transfected with VSVG-rabip4’ for another day and labeled for δ-adaptin and rabip4’. Rabip4’ distribution did not depend on AP-3. Scale bar is 10 µm. Western blots were probed with antibodies against δ-adaptin. The level of δ-adaptin in siRNA-transfected cells was quantified and expressed as % of control (**C**).

The colocalization of the complex on discrete structures (Figure 6B, C) suggests that rabip4’ and AP-3 interact on the endosomal membrane. To investigate a possible role of ARF1 in the function of the rabip4’*AP-3 complex, we incubated HeLa cells expressing VSVG-rabip4’ with BFA and used fluorescence microscopy to monitor their localization. BFA caused a relatively restricted localization of rabip4’-endosomes in the perinuclear area, from which thin tubules emerged (Figure 7A, arrows). This tubules also contain TfR (Figure 7A, inset), supporting their endosomal origin [32], [33]. BFA also redistributed AP-3 from membrane into the cytoplasm in non-transfected and VSVG-rabip4’-expressing cells (Figure 7A), suggesting that the interaction is downstream of ARF1. To investigate if rabip4’ is needed for AP-3 localization to endosomes and vice versa, we efficiently knocked down rabip4s (>85%) or AP-3 (>98%) in HeLa cells (Figure 7) and analyzed the effect on each other distribution by confocal fluorescence microscopy. Neither immunolabeling of AP-3 (Figure 7B) nor of VSVG-rabip4’ (Figure 7C) was affected by the knock-down of rabip4s and AP-3, respectively. Collectively, the results of BFA and the rabip4s/δ-adaptin knock-down experiments show an ARF-dependent, rabip4’-independent localization of AP-3 to endosomes.

### AP-3*rabip4’ Complex Controls Lysosome Distribution via a New Mechanism

**Figure 8:**
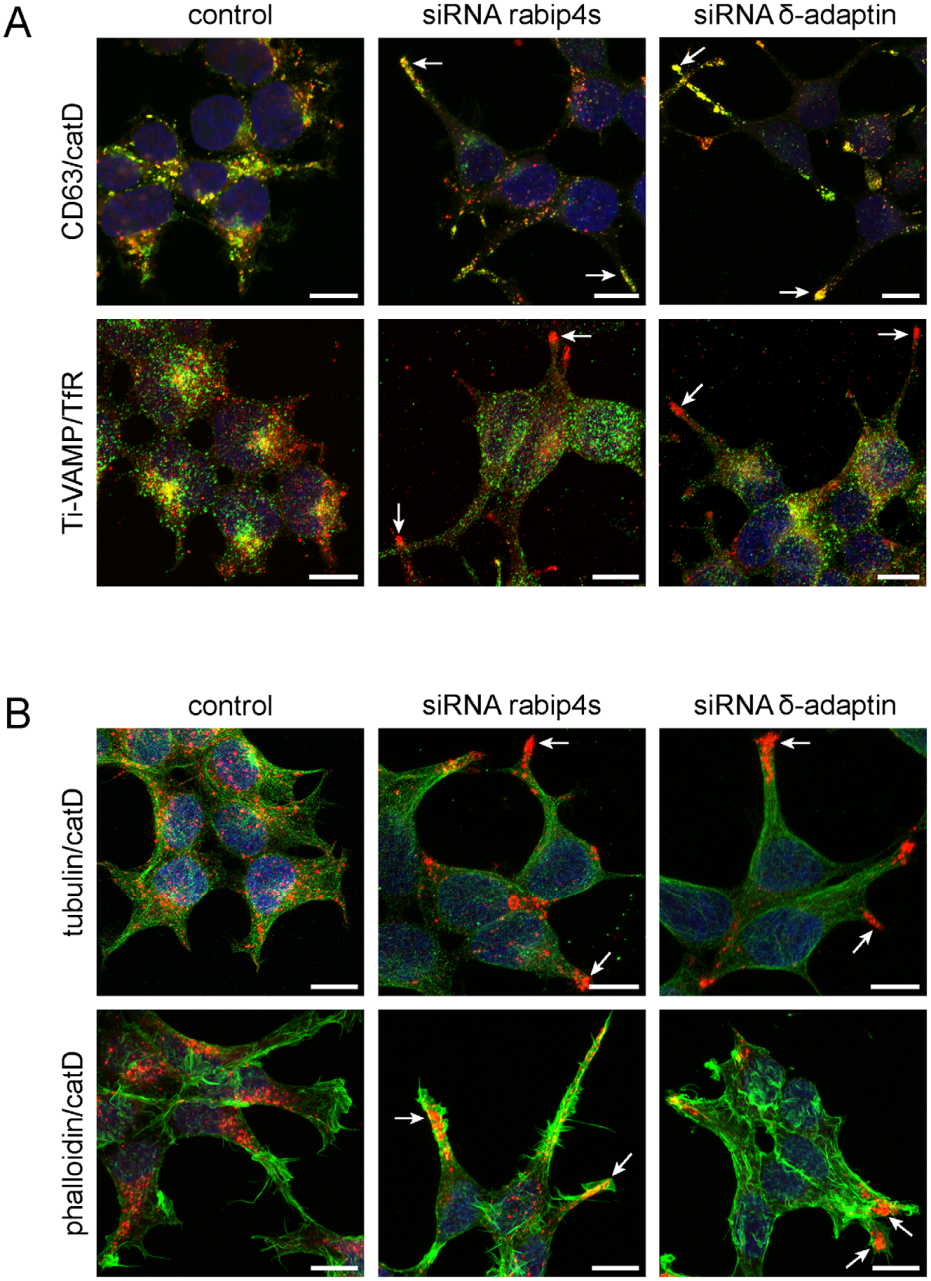
Rabip4’ and AP-3 cooperate in lysosome positioning. HEK293T cells were depleted of rabip4s or AP-3 and then labeled for immunofluorescence with mouse antibodies against CD63 and TfR (green) or with rabbit antisera specific for cathepsin D and Ti-VAMP (red) (**A**). HEK293T cells were processed as above and co-labeled with anti-cathepsin D (red) and anti-tubulin (green) antibodies or with anti-cathepsin D antibody (red) and Alexa-488-conjugated phalloidin for actin staining. Nuclei were stained with DAPI. (**B**). Images represent projections of confocal Z-stacks. Scale bar, 10 µm. Depletion of rabip4s and AP-3 selectively redistributed the lysosomal markers CD63, Cathepsin D, and Ti-VAMP to cellular protrusions (arrows).

The interaction between rabip4’ and AP-3 suggests that they could function in the same intracellular pathway. If so, the CD63 and LAMP-1 phenotype observed in cells depleted of rabip4s should be phenocopied in the absence of AP-3. We therefore knocked down AP-3 in HEK293T cells and followed the intracellular distribution of CD63 and LAMP-1. As in cells depleted of rabip4s, knock-down of AP-3 (Figure 3C and 8A) caused CD63 (Figure 8A) and LAMP-1 (not shown) clustering at the tips of cellular protrusions. This phenotype was more pronounced for AP-3 knock-down, where 75% of cells showed this phenotype, as opposed to 55% in cells silenced for rabip4s and 15% in controls. We next addressed the specificity of AP-3 with respect to the distribution of cargo proteins to the tips of the protrusions in AP-3- or rabip4s-depleted cells. We determined the distributions of Ti-VAMP, a late endosomal/lysosomal v-SNARE that is regulated by AP-3 [22], and TfR, which is AP-3-independent. While Ti-VAMP was present in the protrusions, TfR was excluded from these regions (Figure 8A). The re-distribution of CD63, LAMP-1, and Ti-VAMP to protrusions could either reflect a perturbed intracellular transport of these proteins or a deficiency in lysosome trafficking. To distinguish between these two possibilities, we analyzed the distribution of cathepsin D, of which a precursor is transported independent of AP-3 to lysosomes where it matures by proteolytic cleavage [34]. As shown in Figure 8A, cathepsin D was also re-distributed to cellular protrusions after knock-down of rabip4s or AP-3, showing that the altered distribution of the markers is due to lysosome repositioning and not to a sorting defect. Collectively, these results show that AP-3 and rabip4s function together in a novel pathway that controls the intracellular distribution of lysosomes.

Lysosomes undergo both anterograde and retrograde movement along microtubules, powered by members of the kinesin superfamily and cytoplasmic dynein, respectively [35]. Since efficient lysosome transport requires coordination between the microtubule and actin cytoskeleton [36], we examined the relationship of peripheral lysosomes upon rabip4s or AP-3 depletion with microtubules and F-actin. In cells depleted of rabip4s or AP-3, lysosomes were at the plus end of microtubules, with the most peripheral ones even beyond microtubules, in the cortical actin network (Figure 8B). Since lysosomes in control cells were present along microtubule tracks, this suggested either an accelerated anterograde transport or a deficiency in retrograde, dynein-mediated transport. Dynein recruitment to lysosomes requires the rab7 effector RILP that depends on the activated form of this GTPase for lysosomal localization [9], [12]. Because rab7 was associated with clustered peripheral lysosomes in rabip4s- or AP-3-depleted cells (not shown), we reasoned that these cells had the prerequisite for the localization of the motor complex, but the actual movement of lysosomes along microtubules and perhaps also the loading of lysosomes from the actin filaments to microtubules required rabip4s and AP-3.

AP-3-deficient cells show defects in sorting of LAMPs to lysosomes, which results in their increased trafficking via the plasma membrane [17], [21], [37], [38]. We next determined whether rabip4s also function in the AP-3 pathways to lysosomes. HEK293T cells were depleted of rabip4s or AP-3 (positive control) and cell surface expression of CD63, LAMP-1, and TfR was assayed by flow cytometry (Figure S1). While AP-3 knock-down caused an increase in plasma membrane localization of CD63 and LAMP-1 by more than 2-fold, this shift in the localization was much modest in the absence of rabip4s (Figure S1A, B). Interestingly, although not significant for CD63, depletion of both AP-3 and rabip4s was accompanied by a nearly 2-fold reduction in the total amount of LAMP-1 (Figure S1A, C). Silencing of rabip4s did not substantially affect recycling of TfR (Figure S1A, B), suggesting that it is not an essential regulator of Tf pathways through the endosomal system, perhaps because other rab4 effectors compensate for its absence.

### Rab4 Controls Colocalization of Rabip4’ and AP-3

**Figure 9:**
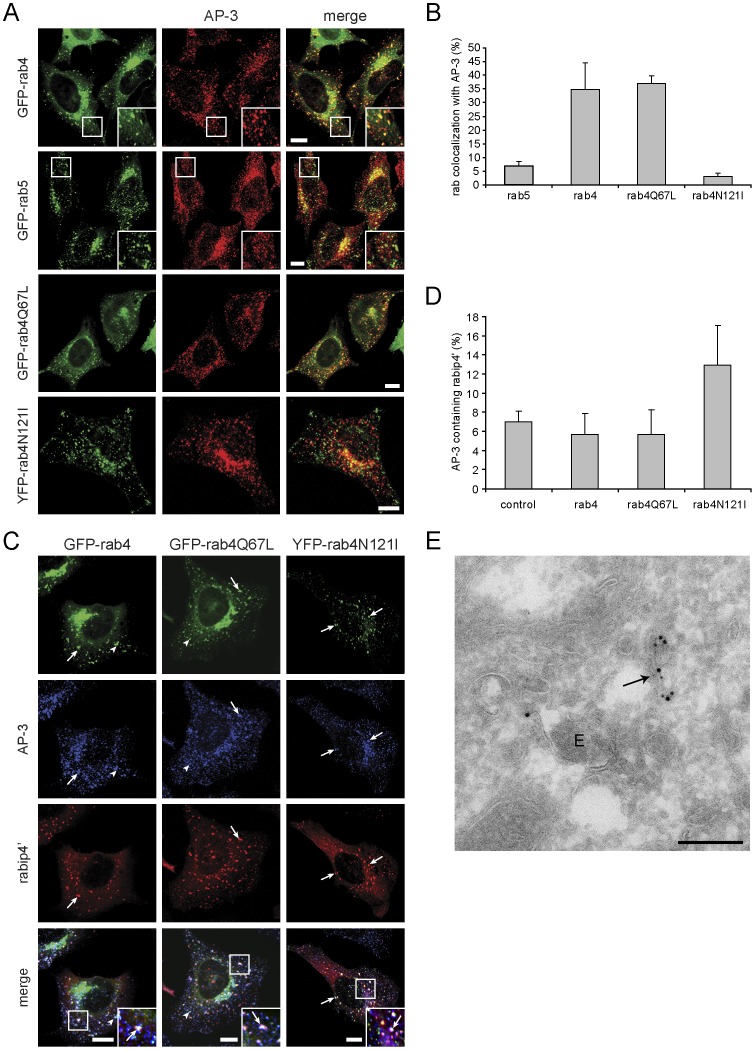
Rab4 regulates the rabip4’*AP-3 complex. HeLa cells expressing the indicated rab4 and rab5 constructs (green) were labeled with a monoclonal antibody against δ-adaptin (red). Rab4 localized with AP-3 predominantly on more peripheral endosomes, while rab5 did not (insets) (**A**). The extent of overlap between rab4 or rab4 mutants and AP-3 was quantified and showed that it was dependent on the GTP-bound form of rab4 (**B**). HeLa cells were co-transfected with VSVG-rabip4’ and the indicated rab4 constructs, and labeled for rabip4’ with a rabbit antibody (red) and δ-adaptin with a mouse antibody (blue). Rab4, rabip4’, and AP-3 colocalized in the perinuclear area independent of the nucleotide status of rab4 (insets, arrows). Active rab4 and AP-3 colocalized on endosomes closer to the cell periphery (arrowheads). Scale bar is 10 µm (**C**). The degree of colocalization between AP-3 and rabip4’ in the absence (control) and in the presence of rab4 or rab4 mutants was quantified. Expression of rab4N121I induced a 2-fold increase in colocalization between rabip4’ and AP-3. Scale bar is 10 µm (**D**). Ultrathin cryosections of HeLa cells transfected with VSVG-rabip4’ and rab4S22N were triple-immunogold labeled for AP-3 (15 nm gold), VSVG (10 nm gold), and rab4 (5 nm gold, indicated by arrows). AP-3 and rabip4’ colocalize on typical recycling tubules (arrow) in the vicinity of endosomal vacuoles (E). Bars, 200 nm (**E**).

Since AP-3 binds rabip4’ adjacent to the rab5 and rab4-interacting domain, we next investigated whether the rabip4’*AP-3 association could be regulated by rab5 and/or by rab4. Rab5 transfection did not affect AP-3 distribution as compared to non-transfected cells (Figure 6A) and little colocalization was seen on peripheral endosomes (Figure 9A, inset GFP-rab5 panel). In contrast, rab4 overexpression decreased the perinuclear staining of AP-3 and increased the size of peripheral endosomes, where rab4 and AP-3 colocalized (Figure 9A, inset GFP-rab4 panel). The extent of colocalization between rab4 and AP-3 was independent of endogenous rabip4’, as it persisted in cells in which rabip4’ was knocked down by RNAi (not shown). We found that 35% of rab4 and 5% of rab5 colocalized with AP-3 on peripheral endosomes (Figure 9A, B). Quantitation of fluorescence was based on signals in the peripheral cytoplasm where discrete structures of GFP-rab5 or rab4 and AP-3 could be distinguished.

The alteration of AP-3 distribution was not due to an interaction with rab4. In pull-down assays with GST-rab4 and rescued *mocha* cell extract, we did not detect binding of AP-3 to GST-rab4 or GST-rab5 (Figure S2). Under these conditions, rabaptin-5α binds to both rabs and can bridge rab4 and AP-1 in a ternary complex [39], [40]. To further explore the role of rab4 in AP-3 localization to a peripheral endosomal population, we expressed constitutively active rab4Q67L and dominant negative rab4N121I mutants, and analyzed AP-3 distribution (Figure 9A). No differences were seen in AP-3 distribution between cells expressing rab4 or rab4Q67L, of which ∼35% colocalized with AP-3 (Figure 9A, B). In cells transfected with rab4N121I, AP-3 localization was the same to that seen in non-transfected cells (Figure 6A) and colocalization with rab4 was lost (Figure 9A, B), arguing that the effect of rab4 on AP-3 depends upon rab4 activation.

We next examined the effect of rab4 on rabip4’-AP-3 colocalization. Cells were co-transfected with rabip4’ and either rab4, rab4Q67L, and rab4N121I (Figure 9C). In double transfectants, we distinguished two endosomal populations: one containing rab4, rabip4’, and AP-3 (arrows, insets) and a second containing AP-3 and rab4 or rab4Q67L (arrowheads). Whereas the endosomes that contained rab4, rabip4’, and AP-3 were located mainly perinuclearly, those positive for rab4 and AP-3 were often found closer to the cell periphery. The perinuclear staining of AP-3 was reduced in cells co-transfected with rabip4’ and rab4 or rab4Q67L, similar to single rab4 and rab4Q67L transfectants. In cells co-expressing rabip4’ and rab4N121I, AP-3 retained its perinuclear localization, with no obvious increase in cell periphery labeling. Many small endosomes that contain only AP-3 were noted (Figure 9C). Quantitation of colocalization between AP-3 and rabip4’ yielded a 2-fold increase in the presence of rab4N121I compared to rab4 or rab4Q67L (Figure 9D). Possibly, binding of rab4GTP to rabip4’ occludes the AP-3 binding site. Since inactive rab4 does not bind rabip4’ [25], its expression will not affect the association of AP-3 with rabip4’. The extent of co-immunoprecipitation of rabip4’ and AP-3 was not affected by transfection of constitutively active or dominant negative rab4 mutants (not shown), suggesting that rab4N121I might increase the residence time of the rabip4’*AP-3 complex on endosomes. In cells transfected with rab4S22N, we found VSVG-rabip4’and AP-3 on recycling tubules in the vicinity of endosomal vacuoles by immunoelectron microscopy (Figure 9E). These results suggest that rab4 acts as a negative regulator of rabip4’-AP-3 interaction, possibly through a competitive binding of rab4 and AP-3 to rabip4’, given the close proximity of rab4 and AP-3 binding sites on rabip4’.

## Discussion

The dynamic localization of organelles within the cytoplasm is a distinguishing feature of cellular organization. Localization contributes to the exchange of content between compartments, and also communication of the cell with its environment. The cytoskeleton is critically involved in this process since it allows for tethering or motor-based movement of organelles. In this study we identified a new complex between AP-3 and rabip4’, and found that they regulate coordinately the spatial distribution of lysosomes downstream of rab5 and rab4. Knock-down of AP-3 or of rabip4’ and its isoform rabip4 causes the accumulation of lysosomes at the end of microtubules, in the peripheral cytoplasm. Rab5 and PI3P are essential determinants for endosomal recruitment of rabip4’, while rab4 regulates the localization of AP-3 and rabip4’ to the same endosomal domain.

### Rab5 and PI(3)P are Upstream Regulators of Rabip4’ Localization

The FYVE domain of rabip4’ is necessary but not sufficient for localization to endosomes [25], suggesting that additional information for endosomal localization is contained elsewhere in rabip4’. Since rabip4’ binds rab5, we reasoned that rab5 was the missing factor for endosomal rabip4’ localization. In the bivalent FYVE domain effector rabenosyn-5, only rab5 is needed for its localization [41]. In accord with this notion, very little rabenosyn-5 associates with endosomal domains harboring rab4 and lacking rab5 [42]. Likewise, endosomal recruitment of rabip4’ only required rab5, whereas the contribution of rab4 was negligible (Figure 2). These findings suggest a common recruitment mechanism for the bivalent rab5-rab4 effectors and the maintenance of vectoriality through the pathway. The interaction of effectors with upstream rab5, assisted by cooperative binding to other factors such as PI(3)P, creates the environment for the formation of a complex with downstream GTPase, i.e. rab4, regulating distal aspects of the pathway. The inability of rab4 to recruit rabip4’ simply reflects the sophistication of the system to maintain directionality of the flow of membrane through the pathway by preventing reverse transport using the same components. Yamamoto et al. recently reported that rabip4 interacts with rab14, but its function in conjunction with rab5-dependent rabip4’ recruitment is not understood and remains to be explored [29].

### Rabip4’ and the Formation of the AP-3 Endosomal Subdomain

Our data uncovered that the FYVE domain of rabip4’ not only is needed for the localization of rabip4’ to PI(3)P-enriched endosomal subdomains (Figure 2), but also binds the AP-3 complex. Importantly, studies of Hoflack et al. show that AP-3 binds PI(3)P-liposomes, which is enhanced by peptides derived from cytoplasmic tails of AP-3 cargo proteins [43]. Conceivably, AP-3, rabip4’, and PI(3)P specify an exit domain on endosomal membrane for certain cargo molecules. The fact that depletion of rabip4s did not result in significant increase of the AP-3 cargoes CD63 and LAMP-1 over the plasma membrane (Figure S1) is consistent with their entrapment earlier in the pathway in the absence of rabip4s.

AP-3 localizes to a tubular endosomal subdomain involved in tyrosinase sorting to melanosomes that is functionally distinct from the endosomal AP-1 domain [20]. Such AP-3 domains occur as well in non-specialized cells, where they mediate specific sorting of cargoes to lysosomes [21]. The observation that rabip4’ and AP-3 localize on the same tubular recycling endosomes (Figure 9E) strongly suggests that rabip4’ contributes to the formation and identity of an endosomal domain selective for AP-3 cargo proteins. Additional evidence for this model comes from the finding that the FYVE domains of EEA1 and Hrs did not bind AP-3, especially since these FYVE proteins regulate other endosomal transport pathways, namely fusion of early endosomes and maturation of MVBs, respectively. The specific interaction of the rabip4’ FYVE domain with AP-3 also shows that the R(R/K)HHCR motif required for PI(3)P binding is not involved in AP-3 binding and that a FYVE domain can also act as protein-protein interaction module.

### Interorganellar Regulation of Lysosome Positioning by AP-3 and Rabip4’

The redistribution of lysosomes in rabip4s- or AP-3-depleted cells resembles the phenotype seen after knock-down of dynein heavy chain [44]. Although lysosomes are not found in cellular protrusions in that case, they do migrate towards the plasma membrane where they organize in patches. Because rabip4’ and AP-3 localize primarily to early/recycling endosomes (this study, [20], [21]), they are unlikely directly involved in the recruitment of the dynein-dynactin complex to lysosomes. Instead, this is a function of the rab7*RILP complex. How could the AP-3*rabip4’ and the rab7*RILP complexes be spatially and functionally linked? In yeast, AP-3 binds vps41 [45], a subunit of the class C Vps/HOPS complex [46], [47] required for tethering of AP-3 positive vesicles to the vacuole [48]. Interactions between AP-3 and mammalian HOPS subunits have also been detected [49]. In mammalian cells, the HOPS complex interacts with rab5 on endosomes and is required for the conversion of rab5 to rab7 that marks endosome maturation [50]. In turn, the HOPS complex functions as an effector of rab7 [51], and the interaction with the Mon1 (SAND1)-Ccz1 complex activates rab7 [52], [53]. Interestingly, in preliminary experiments we localized rab7 together with CD63 in cellular protrusions of AP-3- and, to a lesser extent, of rabip4s-depleted cells. Possibly, depletion of AP-3 and rabip4s prevents activation of rab7, which is consistent with a role of AP-3 and rabip4s upstream of rab7 and RILP in regulating lysosome positioning to the cell center. It is also possible that rabip4s and AP-3 function as motor adaptors, interacting with dynein or another motor protein present on endosomal tubulo-vesicles prior to fusion with lysosomes. Precipitation of dynein heavy chain with immobilized GST-rabip4’ (Figure 4A) supports this possibility, especially in light of interactions between the AP-1 adaptor complex and several kinesins that regulate positioning or transport of TGN [54], [55] and recycling endosomes [56].

The effect of AP-3 and rabip4s depletion on the cytoplasmic position and on the size of lysosomes resemble phenotypes associated with AP-3A deficiency in CTL of HPS2 patients [18]. Secretory lysosomes in HPS2 CTL are unable to move along microtubules to the immunological synapse and do not polarize towards a target cell. In HEK293T cells depleted of AP-3 or rabip4s, the most peripheral lysosomes are found beyond microtubules, suggesting that AP-3 and rabip4s might facilitate initial docking of lysosomes onto microtubules, perhaps from cortical actin filaments, and subsequent movement of lysosomes towards the cell center. The movement of lysosomes to the cell periphery is a prerequisite for lysosomal secretion, which controls such functions as exosome release [57], plasma membrane repair [3], and cell migration [58]. The polarized distribution of microtubules is thought to play a key role in the delivery of new membrane to areas of growth [3]. The formation of plasma membrane projections and polarization of lysosomes towards the tips after AP-3 and rabip4s silencing suggest that these proteins regulate a signaling pathway for cell migration in HEK293T cells. This process possibly implicates the AP-3 cargo protein Ti-VAMP through the Longin domain, since expression of this domain prevents migration of epithelial cells [58].

### Rab4 as a Regulator of Rabip4’-AP-3 Interaction

Rab4 expression affects AP-3 distribution and modulates its colocalization with rabip4’. Rab4 is also known to serve as a docking site for rabaptin5*AP-1 on endosomes where the rab4*rabaptin5*AP-1 axis regulates recycling of TfR [39]. The rab4*rabip4’*AP-3 network we uncovered here functions in a different manner since rab4GTP and AP-3 colocalization does not involve rabip4’. Expression of rab4Q67L generates clusters of small vesicles [59] that could preclude the formation of the AP-3 carriers and cause the redistribution of AP-3 from the perinuclear area to the periphery. A rab4 dominant negative mutant did not affect AP-3 distribution, but increased the colocalization between rabip4’ and AP-3 by 2-fold, suggesting that rab4 is a negative regulator of rabip4’*AP-3 association and that rab4N121I might increase the residence time of the rabip4’*AP-3 complex on endosomes.

A simple model poses that rab4GTP and rabip4’ interact on endosomes and that ongoing GTP hydrolysis generates rab4GDP which dissociates from rabip4’ [25], making the latter available for interaction with AP-3. The adjacent localization of binding sites for rab4 and AP-3 are consistent with a scenario in which steric hindrance prevents their simultaneous association with rabip4’. In conclusion, we discovered a novel complex consisting of AP-3 and rabip4’ that acts downstream of rab5 and rab4 and is a key regulator of lysosomal distribution.

## Experimental Procedures

### Cell Culture, Transfection, and RNAi

HeLa, HEK293T, and rescued *mocha* fibroblasts were grown and transfected as described [21], [39]. siRNA duplexes targeting rabip4s (siRNA ID 32580) and δ-adaptin (ID 137394) or control siRNA were from Ambion (Austin, TX). HeLa and HEK293T cells were transfected with 40 nM siRNA using Lipofectamine RNAiMAX (Invitrogen) and experiments were carried out 3 days post-transfection. Extent of knock-down was determined by Western blot and quantitated using an Odyssey infrared imaging system (LI-COR Biosciences, Lincoln, NE).

### Plasmids and Antibodies

Rab expression constructs have been described [60], [61], [62]. Point mutations were generated by site-directed mutagenesis using the Quick Change kit (Stratagene, La Jolla, CA). pEGFP-rab4 and pEGFP-rab4Q67L were a generous gift of M. Cormont (Nice University, Nice). pmiw-Hrs-HA and pGEX-Hrs-FYVE were from S. Urbé (Physiological Laboratory, Liverpool), and pGEX-EEA1-FYVE was from H. Stenmark (University of Oslo, Oslo). cDNAs encoding AP-3 subunits were kindly provided by M.S. Robinson (CMR, Cambridge), V. Faundez (Emory University, Atlanta), and J.S. Bonifacino (NIH, Bethesda) and subcloned in pcDNA3 or pCIneo. β3A constructs were cloned in pGlo-Myc [39]. Rabip4’ΔCC3 lacking aa 524–633 was generated by overlap-extension PCR. Amplified cDNAs were verified by DNA sequencing. Antibody #444 against a common epitope in rabip4s was generated by immunizing rabbits with GST-rabip4’(aa 509–708). Mouse anti-VSVG, rabbit anti-EEA1 [25], rabbit anti-rab4, and rabbit anti-rabaptin-5 [39] antibodies were described in the indicated references. Rabbit antibody against β3A and mouse anti-δ-adaptin were generously provided by M.S. Robinson and A. Peden (CMR, Cambridge), respectively. Rabbit antiserum against Ti-VAMP was a gift from T. Galli (INSERM, Paris). Cathepsin D antibodies raised in rabbits were provided to us by A. Hasilik (University of Marburg, Marburg) and S. Kornfeld (Washington University School of Medicine, St Louis) and used for immunofluorescence microscopy and Western blot, respectively. The following mouse antibodies were purchased from commercial sources: 2G11 anti-CI-MPR, H5G11 anti-LAMP-1, MX-49.129.15 anti-CD63, H68.4 (Santa Cruz Biotechnology) and OKT9 (ATCC, Rockville, MD) anti-TfR, anti-β3B (β-NAP), anti-μ3A (p47A), anti-σ3A, anti-γ1-adaptin, anti-EEA1 (BD Biosciences, San Jose, CA), 100/3 against γ1-adaptin, 100/1 against α-adaptin, 100/2 against β1,2-adaptin (Sigma), AP-6 against α-adaptin (ABR, Breda, NL), C4 anti-actin (ICN Biomedicals, Costa Mesa, CA), anti-tubulin (Invitrogen). Other antibodies used in this study were: rabbit anti-VSVG (Bethyl Inc., Montgomery, TX), rabbit anti-HA (Sigma), affinity purified rabbit anti-mouse IgG (Jackson Immunoresearch Laboratories, West Grove, PA), HRP-labeled secondary antibodies (Jackson Immunoresearch Laboratories), and fluorescently labeled secondary antibodies (Invitrogen).

### Binding Assays

Pull-down assays with GST fusion proteins and cytosol or cell extracts were done as described [60], [61], [62]. Bound proteins were eluted with 1.5 M NaCl or boiled off in reducing Laemmli sample buffer and analyzed by Western blotting. Eluates of preparative pull-downs with GST-rabip4’(aa 299–708) and cytosol were analyzed by LC-MS/MS as before [62]. AP-3 subunits and β3A truncations were produced by *in vitro* transcription-translation in the presence of ^35^S-methionine and directly used in binding assays as described [62]. Bound proteins were resolved by SDS-PAGE and analyzed by phosphorimaging. Hela cells expressing VSVG-rabip4’ were washed with ice-cold PBS, lysed in 50 mM HEPES pH 7.4, 150 mM NaCl, 1.5 mM MgCl_2_, 1 mM EDTA, 1% Triton X100, and protease inhibitors. Lysates were subjected to immunoprecipitation as described [63]. Bound proteins were separated by SDS-PAGE and analyzed by Western blot.

### Fluorescence Microscopy and Image Processing

Cells were grown to ∼50% confluency on coverslips. After 2 days, cells were washed with PBS, fixed in 3% paraformaldehyde, 100 mM phosphate buffer pH 7.4 for 30 min at room temperature and processed for immunofluorescence microscopy as described [64]. When indicated, cells were treated with 100 nM PI3-kinase inhibitor wortmannin (Sigma) for 15 min at 37°C or with 5 µg/ml brefeldin A (Sigma) for 15 min at 37°C and processed as described above. Alexa488-conjugated Phalloidin (Invitrogen) was used to detect F-actin. Coverslips were mounted in Mowiol or Prolong Gold anti-fade with DAPI (Invitrogen) and examined with a Zeiss-LSM-710 confocal microscope (Carl Zeiss, Weesp, The Netherlands). For quantification experiments, 10 cells from two independent transfections were analyzed. Quantification of signal overlap was performed using MetaMorph (Universal Imaging, Downingtown, PA). Evaluation of rabip4’, EEA1, and Hrs relative distribution was done manually. Briefly, the endosomes that contained rabip4’ were counted and set to 100%. The number of rabip4’-positive endosomes that labeled also for EEA1 and Hrs was then expressed as percentage of rabip4’-endosomes. Quantification of RNAi phenotype was done as follows: 6 random fields from 2 independent experiments were analyzed and the number of cells with overgrown cellular protrusions and redistribution of lysosomes to these protrusions at the expense of the cell body was expressed as percentage of the total (n = 108 cells in control, 97 cells in rabip4s RNAi, and 99 cells in AP-3 RNAi).

### Immunoelectron Microscopy

HeLa cells expressing rab4S22N and VSVG-rabip4’ were fixed by adding 4% freshly prepared formaldehyde or a mixture of 0.2% glutaraldehyde and formaldehyde in 0.1 M phosphate-buffer, pH 7.4, to an equal volume of culture medium. Cells were then prepared for ultrathin cryosectioning and immunogold labeled according to the protein A-gold method [65].

### Flow Cytometry

HEK293T cells transfected with control siRNA or targeting AP-3 or rabip4s were detached from the plates using 0.02% EDTA in PBS. Cells were resuspended in FACS buffer (2% fetal calf serum in PBS). Approximately 10^5^ cells were distributed in FACS tubes and washed once with the same buffer. Before staining, cells were either (i) fixed with 1% PFA for 10 min followed by 0.25% saponin permeabilization for total expression quantification or (ii) kept on ice for cell surface determination. Primary antibodies were added in saponin/FACS buffer and samples were incubated for 30 min at room temperature/on ice for staining of total/surface expression, respectively. Cells were washed in saponin/FACS buffer and incubated for 20 min with Alexa Fluor (AF) 488-conjugated secondary antibodies. Fluorescence intensity was determined using a FACSCalibur instrument (BD Biosciences) for 10,000 acquired cells. Two independent experiments in duplicate were performed. Statistical analysis was performed with Cell Quest Pro Software. All data are represented as average of the median fluorescence intensities (MFI) ± SD after normalization to siRNA control values.

## Supporting Information

Figure S1 Rabip4s have minimal function in transport to lysosomes. Control, rabip4s-, and AP-3-depleted cells were harvested and processed for flow cytometry as described in experimental procedures for surface and total staining of CD63, LAMP-1, and TfR (**A** and **B**). FACS profiles of the cell surface and total expression of the indicated cargo proteins in control (pink line), rabip4s- (purple, filled histogram), and AP-3-depleted cells (green line) (**A**). The cell surface over total expression levels were quantified and values were normalized to controls. Rabip4s knock-down minimally affects the cell surface appearance of CD63, LAMP-1, and TfR (**B**). Control, rabip4s-, and AP-3-depleted cells were lysed and subjected to SDS-PAGE and Western blot with the indicated antibodies. Both rabip4s and AP-3 knock-down reduced the total amount of LAMP-1 (**C**).
(TIF)

Figure S2 Rab4 does not bind AP-3. GST, GST-rab4, and GST-rab5 were isolated on GSH beads. GST-rabs were loaded with either GMP-PNP (the non-hydrolysable GTP analog and referred to as GTP for simplicity) or GDP and incubated with rescued *mocha* cell lysate. Bound fractions were immunoblotted with antibodies against δ-adaptin and rabaptin-5α. AP-3 did not bind to rab4 or rab5.
(TIF)

## References

[1] Luzio JP, Pryor PR, Bright NA (2007) Lysosomes: fusion and function. Nature Revs Mol Cell Biol 8: 622–632. 17637737 10.1038/nrm2217

[2] Saftig P, Klumperman J (2009) Lysosome biogenesis and lysosomal membrane proteins: trafficking meets function. Nature Revs Mol Cell Biol 10: 623–635. 19672277 10.1038/nrm2745

[3] Idone V, Tam C, Andrews NW (2008) Two-way traffic on the road to plasma membrane repair. Trends Cell Biol 18: 552–559. 18848451 10.1016/j.tcb.2008.09.001 PMC2593466

[4] Huizing M, Helip-Wooley A, Westbroek W, Gunay-Aygun M, Gahl WA (2008) Disorders of lysosome related organelle biogenesis: clinical and molecular genetics. Ann Rev Genom Hum Mol Genet 9: 359–386. 10.1146/annurev.genom.9.081307.164303 PMC2755194 18544035

[5] Schmid JP, Cote M, Menager MM, Burgess A, Nehme N, et al (2010) Inherited defects in lymphocyte cytotoxic activity. Immunol Rev 235: 10–23. 20536552 10.1111/j.0105-2896.2010.00890.x

[6] Brown CL, Maier KC, Stauber T, Ginkel LM, Wordeman L, et al (2005) Kinesin-2 is a motor for late endosomes and lysosomes. Traffic 6: 1114–1124. 16262723 10.1111/j.1600-0854.2005.00347.x

[7] Hendricks AG, Perlson E, Ross JL, Schroeder HWr, Tokito M, et al (2010) Motor coordination via a tug of war mechanism drives bidirectional vesicle transport. Curr Biol 20: 697–702. 20399099 10.1016/j.cub.2010.02.058 PMC2908734

[8] Loubery S, Wilhelm C, Hurbain I, Nevewu S, Louvard D, et al (2008) Different microtubule motors move early and late endocytic compartments. Traffic 9: 492–509. 18194411 10.1111/j.1600-0854.2008.00704.x

[9] Jordens I, Fernandez-Borja M, Marsman M, Dusseljee S, Janssen L, et al (2001) The rab7 effector protein RILP controls lysosomal transport by inducing the recruitment of dynein-dynactin motors. Curr Biol 11: 1680–1685. 11696325 10.1016/s0960-9822(01)00531-0

[10] Tan SC, Scherer J, Vallee RB (2011) Recruitment of dynein to late endosomes and lysosomes through light intermediate chains. Mol Biol Cell 22: 467–477. 21169557 10.1091/mbc.E10-02-0129 PMC3038645

[11] Johansson M, Rocha N, Zwart WT, Jordens I, Janssen L, et al (2007) Activation of endosomal dynein motors by stepwise assembly of rab7-RILP-p150Glued, ORP1L and the receptor betaIIIspectrin. J Cell Biol 176: 459–471. 17283181 10.1083/jcb.200606077 PMC2063981

[12] Cantalupo G, Alifano P, Roberti V, Bruni CB, Bucci C (2001) Rab-interacting lysosomal protein (RILP): the rab7 effector required for transport to lysosomes. EMBO J 20: 683–693. 11179213 10.1093/emboj/20.4.683 PMC145419

[13] Rocha N, Kuijl C, van der Kant R, Janssen L, Houben D, et al (2009) Cholesterol sensor ORP1L contacts the ER protein VAP to control rab7-RILP-p150Glued and late endosome positioning. J Cell Biol 185: 1208–1225. 10.1083/jcb.200811005 PMC2712958 19564404

[14] Rosa-Ferreira C, Munro S (2011) Arl8 and SKIP act together to link lysosomes to kinesin-1. Dev Cell 21: 1171–1178. 22172677 10.1016/j.devcel.2011.10.007 PMC3240744

[15] Hirst J, Barlow LD, Francisco GC, Sahlender DA, Seaman MN, et al (2011) The fifth adaptor protein complex. PLoS Biol 9: e1001170. 22022230 10.1371/journal.pbio.1001170 PMC3191125

[16] Robinson MS, Bonifacino JS (2001) Adaptor-related proteins. Curr Opin Cell Biol 13: 444–450. 11454451 10.1016/s0955-0674(00)00235-0

[17] Dell’Angelica EC, Shotelersuk V, Aguilar RC, Gahl WA, Bonifacino JS (1999) Altered trafficking of lysosomal proteins in Hermansky-Pudlak syndrome due to mutations in the beta3A subunit of the AP-3 adaptor. Mol Cell 3: 11–21. 10024875 10.1016/s1097-2765(00)80170-7

[18] Clark RH, Stinchcombe JC, Day A, Blott E, Booth S, et al (2003) Adaptor protein 3-dependent microtubule mediated movement of lytic granules to the immunological synapse. Nature Immunol 4: 1111–1120. 14566336 10.1038/ni1000

[19] Zhen L, Feng JL, Bright NA, Peden AA, Seymour AB, et al (1999) Abnormal expression and subcellular distribution of subunit proteins of the AP-3 adaptor complex lead to platelet storage pool deficiency in the pearl mouse. Blood 94: 146–155. 10381507

[20] Theos AC, Tenza D, Martina JA, Hurbain I, Peden AA, et al (2005) Functions of adaptor protein (AP)-3 and AP-1 in tyrosinase sorting from endosomes to melanosomes. Mol Biol Cell 16: 5356–5372. 16162817 10.1091/mbc.E05-07-0626 PMC1266432

[21] Peden AA, Oorschot V, Hesser BA, Austin CD, Scheller RH, et al (2004) Localization of the AP-3 adaptor complex defines a novel endosomal exit site for lysosomal membrane proteins. J Cell Biol 164: 1065–1076. 15051738 10.1083/jcb.200311064 PMC2172074

[22] Martinez-Arca S, Rudge R, Vacca M, Raposo G, Camonis J, et al (2003) A dual mechanism controlling the localization and function of exocytic v-SNAREs. Proc Natl Acad Sci USA 100: 9011–9016. 12853575 10.1073/pnas.1431910100 PMC166429

[23] Hutagalung AH, Novick PJ (2011) Role of rab GTPases in membrane traffic and cell physiology. Physiol Rev 91: 119–149. 21248164 10.1152/physrev.00059.2009 PMC3710122

[24] Stenmark H (2009) Rab GTPases as coordinators of vesicle traffic. Nature Revs Mol Cell Biol 10: 513–525. 19603039 10.1038/nrm2728

[25] Fouraux M, Deneka M, Ivan V, van der Heijden A, Raymackers J, et al (2004) rabip4’ is an effector of rab5 and rab4 and regulates transport through early endosomes. Mol Biol Cell 15: 611–624. 14617813 10.1091/mbc.E03-05-0343 PMC329268

[26] Lloyd TE, Atkinson R, Wu MN, Zhou Y, Pennetta G, et al (2002) Hrs regulates endosome membrane invagination and tyrosine kinase receptor signaling in Drosophila. Cell 108: 261–269. 11832215 10.1016/s0092-8674(02)00611-6

[27] Navaroli DM, Bellve KD, Standley C, Lifshitz LM, Cardia J, et al (2012) Rabenosyn-5 defines the fate of the transferrin receptor following clatrin-mediated endocytosis. Proc Natl Acad Sci USA 109: 471–480. 10.1073/pnas.1115495109 PMC3286945 22308388

[28] Cormont M, Mari M, Galmiche A, Hofman P, Le Marchand-Brustel Y (2001) A FYVE finger-containing protein, rabip4, is a rab4 effector protein involved in early endosomal traffic. Proc Natl Acad Sci USA 98: 1637–1642. 11172003 10.1073/pnas.031586998 PMC29309

[29] Yamamoto H, Koga H, Katoh Y, Takahashi S, Nakayama K, et al (2010) Functional cross-talk between rab14 and rab4 through a dual effector RUFY1/rabip4. Mol Biol Cell 21: 2746–2755. 20534812 10.1091/mbc.E10-01-0074 PMC2912359

[30] Blumstein J, Faundez V, Nakatsu F, Saito T, Ohno H, et al (2001) The neuronal form of adaptor protein-3 is required for synaptic vesicle formation from endosomes. J Neurosci 21: 8034–8042. 11588176 10.1523/JNEUROSCI.21-20-08034.2001 PMC6763874

[31] Peden AA, Rudge RE, Lui WY, Robinson MS (2002) Assembly and function of AP-3 complexes in cells expressing mutant subunits. J Cell Biol 156: 327–336. 11807095 10.1083/jcb.200107140 PMC2199225

[32] Daro E, van der Sluijs P, Galli T, Mellman I (1996) Rab4 and cellubrevin define different early endosome populations on the pathway of transferrin receptor recycling. Proc Natl Acad Sci USA 93: 9559–9564. 8790369 10.1073/pnas.93.18.9559 PMC38467

[33] van Dam E, Stoorvogel W (2002) Dynamin-dependent transferrin recycling by endosome-derived clathrin-coated vesicles. Mol Biol Cell 13: 169–182. 11809831 10.1091/mbc.01-07-0380 PMC65080

[34] von Figura K, Hasilik A (1986) Lysosomal enzymes and their receptors. Annu Rev Biochem 55: 167–193. 2943218 10.1146/annurev.bi.55.070186.001123

[35] Caviston JP, Holzbaur EL (2006) Microtubule motors at the intersection of trafficking and transport. Trends Cell Biol 16: 530–537. 16938456 10.1016/j.tcb.2006.08.002

[36] Cordonnier MN, Dauzonne D, Louvard D, Coudrier E (2001) Actin filaments and myosin I alpha cooperate with microtubules for the movement of lysosomes. Mol Biol Cell 12: 4013–4029. 11739797 10.1091/mbc.12.12.4013 PMC60772

[37] Lefrancois S, Janvier K, Boehm M, Ooi CE, Bonifacino JS (2004) An ear-core interaction regulates the recruitment of the AP-3 complex to membranes. Dev Cell 7: 619–625. 15469849 10.1016/j.devcel.2004.08.009

[38] Janvier K, Bonifacino JS (2005) Role of the endocytic machinery in sorting of lysosome-associated membrane proteins. Mol Biol Cell 16: 4231–4242. 15987739 10.1091/mbc.E05-03-0213 PMC1196333

[39] Deneka M, Neeft M, Popa I, van Oort M, Sprong H, et al (2003) rabaptin-5α/rabaptin-4 serves as a linker between rab4 and γ1-adaptin in membrane recycling from endosomes. EMBO J 22: 2645–2657. 12773381 10.1093/emboj/cdg257 PMC156754

[40] Popa I, Deneka M, van der Sluijs P (2005) Expression and properties of the rab4, rabaptin-5alpha, AP-1 complex in endosomal recycling. Meth Enzymol 403: 526–540. 16473617 10.1016/S0076-6879(05)03046-6

[41] Nielsen E, Christoforidis S, Utenwiler-Joseph S, Miaczynska M, Dewitte F, et al (2000) Rabenosyn-5, a novel rab5 effector, is complexed with hVPS45 and recruited to endosomes through a FYVE finger domain. J Cell Biol 151: 601–612. 11062261 10.1083/jcb.151.3.601 PMC2185588

[42] de Renzis S, Sönnichsen B, Zerial M (2002) Divalent rab effectors regulate the sub-compartmental organization and sorting function of early endosomes. Nature Cell Biol 4: 124–133. 11788822 10.1038/ncb744

[43] Baust T, Anitei M, Czupalla C, Parshyna I, Bourel L, et al (2008) Protein networks supporting AP-3 function in targeting lysosomal membrane proteins. Mol Biol Cell 19: 1942–1951. 18287518 10.1091/mbc.E08-02-0110 PMC2366865

[44] Caviston JP, Zajac A, Tokito M, Holzbauer ELF (2010) Huntington coordinates the dynein-mediated dynamic positioning of endosomes and lysosomes. Mol Biol Cell 22: 478–492. 21169558 10.1091/mbc.E10-03-0233 PMC3038646

[45] Darsow T, Katzmann DJ, Cowles CR, Emr SD (2001) Vps21p function in the alkaline phosphatase pathway requires homo-oligomerization and interaction with AP-3 through two distinct domains. Mol Biol Cell 12: 37–51. 11160821 10.1091/mbc.12.1.37 PMC30566

[46] Rieder SE, Emr SD (1997) A novel RING finger protein complex essential for a late step in protein transport to the yeast vacuole. Mol Biol Cell 8: 2307–2327. 9362071 10.1091/mbc.8.11.2307 PMC25710

[47] Wurmser AE, Sato TK, Emr SD (2000) New component of the vacuolar class C-Vps complex couples nucleotide exchange on the Ypt7 GTPase to SNARE dependent docking and fusion. J Cell Biol 151: 551–562. 11062257 10.1083/jcb.151.3.551 PMC2185595

[48] Angers CG, Merz AJ (2010) New links between vesicle coats and rab-mediated vesicle targeting. Sem Cell Dev Biol 22: 18–26. 10.1016/j.semcdb.2010.07.003 PMC3196633 20643221

[49] Zlatic SA, Tornieri K, L’Hernault SW, Faundez V (2011) Clathrin-dependent mechanisms modulate the subcellular distribution of class C Vps/HOPS tether subunits in polarized and nonpolarized cells. Mol Biol Cell 15: 1699–1715. 10.1091/mbc.E10-10-0799 PMC3093322 21411634

[50] Rink J, Ghigo E, Kalaidzidis Y, Zerial M (2005) Rab conversion as a mechanism of progression from early to late endosomes. Cell 122: 735–749. 16143105 10.1016/j.cell.2005.06.043

[51] Seals DF, Eitzen G, Margolis N, Wickner WT, Price A (2000) A Ypt/rab effector complex containing the sec1 homolog vps33p is required for homotypic vacuole fusion. Proc Natl Acad Sci USA 97: 9402–9407. 10944212 10.1073/pnas.97.17.9402 PMC16876

[52] Poteryaev D, Datta S, Ackema K, Zerial M, Spang A (2010) Identification of the switch in early to late endosome transition. Cell 141: 497–508. 20434987 10.1016/j.cell.2010.03.011

[53] Nordmann M, Cabrera M, Perz A, Brocker C, Ostrowicz C, et al (2010) The Mon1-CCz1 complex is the GEF of the late endosomal Rab7 homolog Ypt7. Curr Biol 20: 1654–1659. 20797862 10.1016/j.cub.2010.08.002

[54] Nakagawa T, Setou M, Seog DH, Ogasawara K, Dohmae N, et al (2000) A novel motor, KIF13A, transports mannose 6-phosphate receptor to plasma membrane through direct interaction with AP-1 complex. Cell 103: 568–581. 10.1016/s0092-8674(00)00161-6 11106728

[55] Schmidt MR, Maritzen T, Kukhtina V, Higman VA, Doglio L, et al (2009) Regulation of endosomal membrane traffic by a Gadkin/AP-1/kinesin KIF5 complex. Proc Natl Acad Sci USA 106: 15344–15349. 19706427 10.1073/pnas.0904268106 PMC2741253

[56] Delevoye C, Hurbain I, Tenza D, Sibarita JB, Gafsou SU, et al (2009) AP-1 and KIF13A coordinate endosomal sorting and positioning during melanosome biogenesis. J Cell Biol 187: 247–264. 19841138 10.1083/jcb.200907122 PMC2768840

[57] Simons M, Raposo G (2009) Exosomes-vesicular carriers for intercellular communication. Curr Opin Cell Biol 21: 575–581. 19442504 10.1016/j.ceb.2009.03.007

[58] Proux-Gillardeaux V, Raposo G, Irinopoulou T, Galli T (2007) Expression of the Longin domain of TI-VAMP impairs lysosomal secretion and epithelial cell migration. Biol Cell 99: 261–271. 17288539 10.1042/BC20060097

[59] de Wit H, Lichtenstein Y, Kelly RB, Geuze HJ, Klumperman J, et al (2001) Rab4 regulates formation of synaptic-like microvesicles from early endosomes in PC12 cells. Mol Biol Cell 12: 3703–3715. 11694600 10.1091/mbc.12.11.3703 PMC60287

[60] van Vlijmen T, Vleugel M, Evers M, Mohammed S, Wulf PS, et al (2008) A unique residue in rab3c determines the interaction with novel binding protein Zwint-1. FEBS Lett 582: 2938–2942. 10.1016/j.febslet.2008.07.012 18625232

[61] Rojas R, van Vlijmen T, Mardones G, Mohammed S, AJR H, et al (2008) Regulation of retromer recruitment to endosomes by sequential action of rab5 and rab7. J Cell Biol 183: 513–526. 18981234 10.1083/jcb.200804048 PMC2575791

[62] Hoogenraad CC, Popa I, Futai K, Martinez-Sanchez E, Wulf PS, et al (2010) Neuron specific rab4 effector GRASP-1 coordinates membrane specialization and maturation of recycling endosomes. PLoS Biol 8: e1000283. 20098723 10.1371/journal.pbio.1000283 PMC2808209

[63] Ivan V, de Voer G, Xanthakis D, Spoorendonk KM, Kondylis V, et al (2008) Drosophila Sec16 mediates the biogenesis of tER sites upstream of Sar1 through an arginine-rich motif. Mol Biol Cell 19: 4352–4365. 18614796 10.1091/mbc.E08-03-0246 PMC2555954

[64] Elstak ED, Neeft M, Nehme NT, Voortman J, Cheung M, et al (2011) Munc13–4 rab27 complex is specifically required for tethering secretory lysosomes at the plasma membrane. Blood 118: 1570–1578. 21693760 10.1182/blood-2011-02-339523

[65] Liou W, Geuze HJ, Slot JW (1996) Improved structural integrity of cryosections for immunogold labeling. Histochem Cell Biol 106: 41–58. 8858366 10.1007/BF02473201

