# Supplementary figures and images for: AP-3 and Rabip4’ Coordinately Regulate Spatial Distribution of Lysosomes
Source: PLoS One. 2012 Oct 29;7(10):e48142. doi: 10.1371/journal.pone.0048142 (PMC3483219; doi:10.1371/journal.pone.0048142)

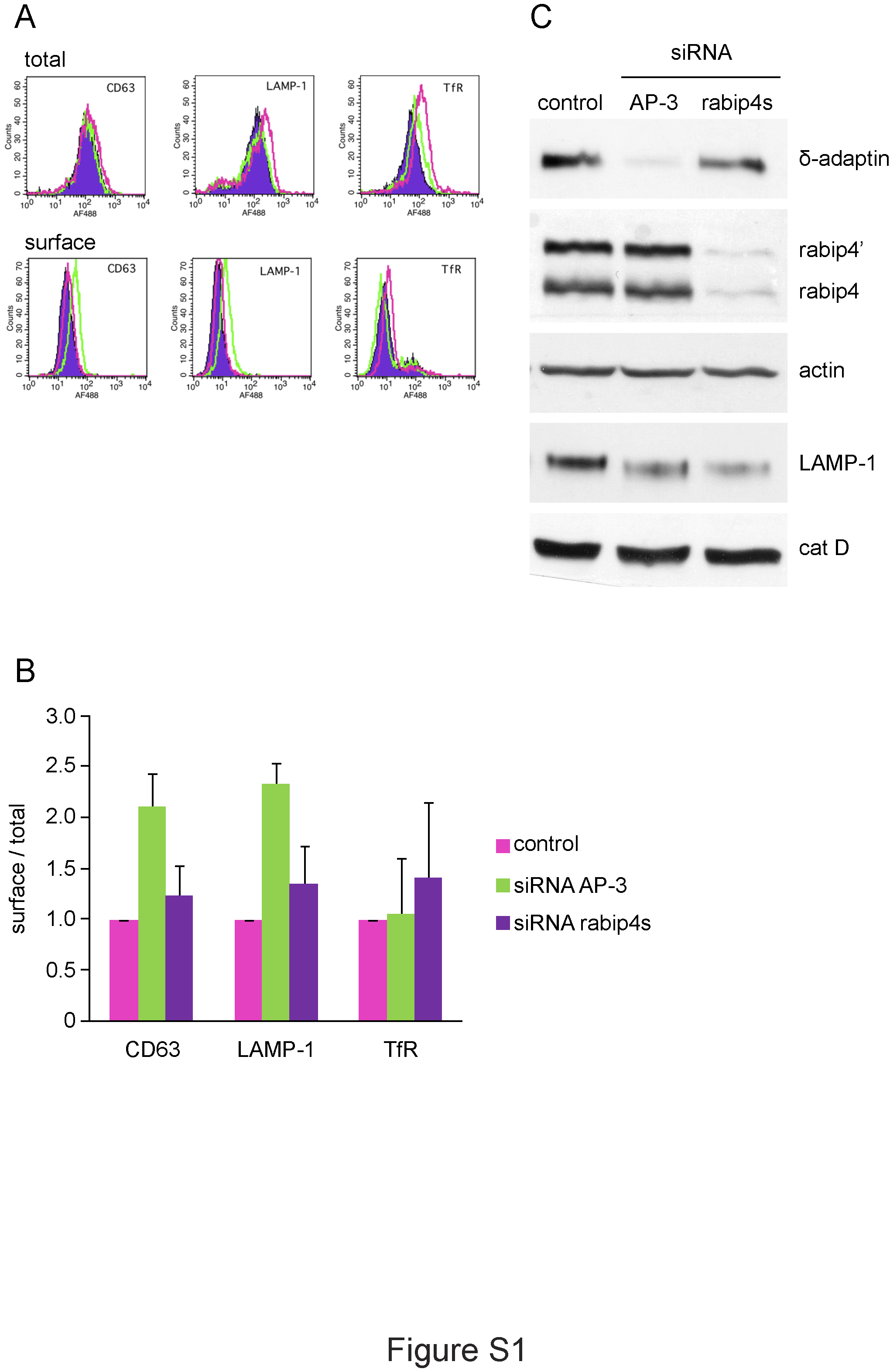

Supplement: Figure S1 — Rabip4s have minimal function in transport to lysosomes. Control, rabip4s-, and AP-3-depleted cells were harvested and processed for flow cytometry as described in experimental procedures for surface and total staining of CD63, LAMP-1, and TfR (A and B). FACS profiles of the cell surface and total expression of the indicated cargo proteins in control (pink line), rabip4s- (purple, filled histogram), and AP-3-depleted cells (green line) (A). The cell surface over total expression levels were quantified and values were normalized to controls. Rabip4s knock-down minimally affects the cell surface appearance of CD63, LAMP-1, and TfR (B). Control, rabip4s-, and AP-3-depleted cells were lysed and subjected to SDS-PAGE and Western blot with the indicated antibodies. Both rabip4s and AP-3 knock-down reduced the total amount of LAMP-1 (C). (TIF) [file pone.0048142.s001.tif]

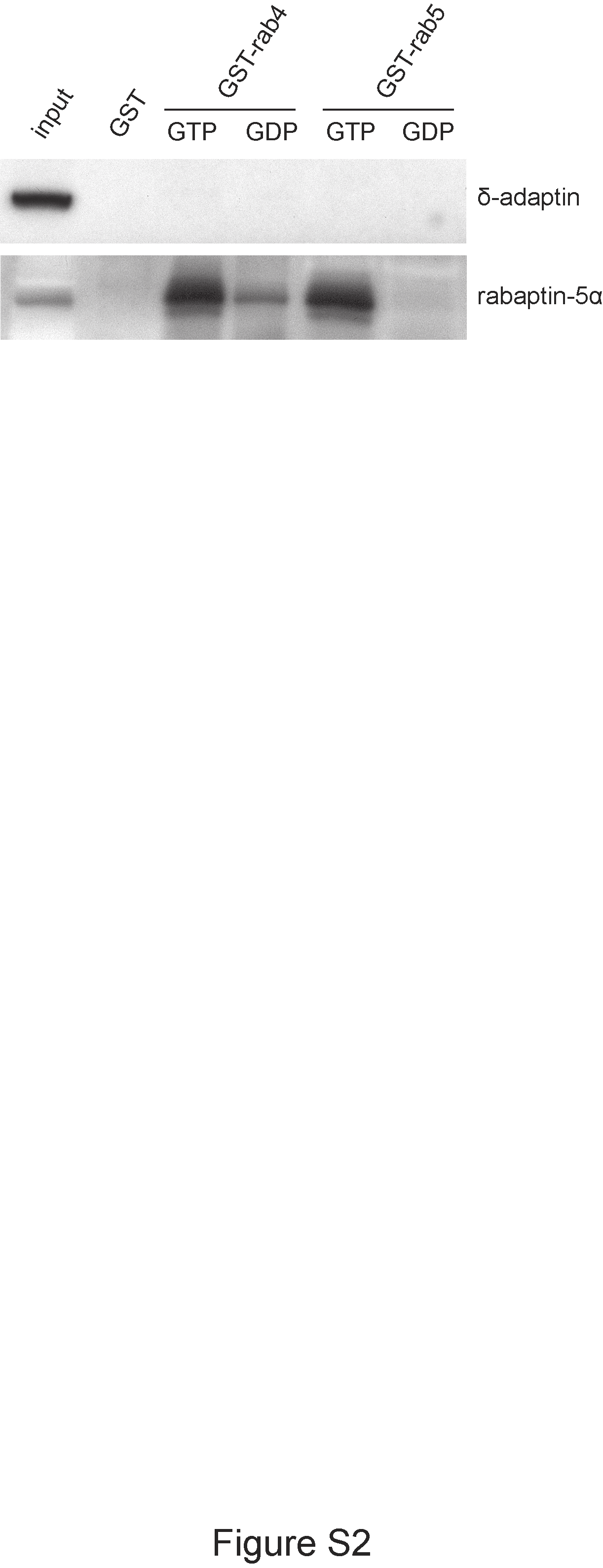

Supplement: Figure S2 — Rab4 does not bind AP-3. GST, GST-rab4, and GST-rab5 were isolated on GSH beads. GST-rabs were loaded with either GMP-PNP (the non-hydrolysable GTP analog and referred to as GTP for simplicity) or GDP and incubated with rescued mocha cell lysate. Bound fractions were immunoblotted with antibodies against δ-adaptin and rabaptin-5α. AP-3 did not bind to rab4 or rab5. (TIF) [file pone.0048142.s002.tif]
